# Comparative Efficacy of Treatments for Radiation-Induced Oral Mucositis in Patients With Head and Neck Cancer

**DOI:** 10.7759/cureus.86145

**Published:** 2025-06-16

**Authors:** Raj Patel, Shareef Amor, Tadas Masys, Jay Patel

**Affiliations:** 1 Otolaryngology - Head and Neck Surgery, Loyola University Chicago Stritch School of Medicine, Chicago, USA; 2 Dentistry, Roseman University of Health Sciences College of Dental Medicine, South Jordan, USA; 3 Biology, Loyola University of Chicago, Chicago, USA; 4 Dentistry, Aegis Dental Group, Indianapolis, USA

**Keywords:** dental treatment outcome, head and neck cancer, oral diseases, outcome measure, radiation-induced oral mucositis

## Abstract

Radiation-induced oral mucositis is a common and painful side effect in patients with head and neck cancer undergoing radiation therapy, causing inflammation and ulceration of the oral mucosa. This condition severely impacts a patient’s ability to eat, speak, or swallow, making effective prevention crucial for improving quality of life. This review aimed to evaluate and compare treatments for radiation-induced oral mucositis, focusing on symptom relief and side effects. We included studies with clear outcome measures, pre- and post-treatment scores, and demographic data, excluding case reports, reviews, and abstracts. Our literature search through PubMed and the Excerpta Medica Database (EMBASE) identified 190 articles, which were narrowed down to nine after abstract analysis. From the systematic review findings, omega-3 nanoemulgel demonstrated the lowest complication rate (2.9%), with minimal impact on symptoms, making it the most effective treatment. In contrast, rebamipide and benzydamine had higher complication rates (25% and 28%, respectively) and less significant symptom relief. Given its safety, efficacy, and minimal side effects, omega-3 nanoemulgel should be considered a first-line preventive treatment for oral mucositis in patients with head and neck cancer.

## Introduction and background

Head and neck cancers represent approximately 3-4% of all cancers diagnosed in the United States [1]. These malignancies arise from specific anatomical sites, including the oral cavity, pharynx, hypopharynx, larynx, nasal cavity, salivary glands, and scalp [2]. Among these, the most common primary sites are the larynx (30.37%), lips and oral cavity (29.08%), pharynx (20.03%), and salivary glands (10.94%) [3]. The vast majority, around 90%, of head and neck cancers are squamous cell carcinomas [4]. Although basal cell carcinoma is the most prevalent skin cancer overall, it is relatively uncommon in the head and neck region, comprising only about 10% of skin cancers in this area [5].

Management of head and neck cancers is often complex and depends on several factors, including tumor stage, histological grade, depth of invasion, and severity of clinical symptoms [6]. Surgical excision remains the primary mode of treatment, frequently followed by adjuvant radiation therapy to enhance local control by targeting residual microscopic disease and achieving clear surgical margins [7]. However, radiation therapy is associated with a range of adverse effects. Systemic side effects may include fatigue, skin irritation, alopecia, nausea, vomiting, diarrhea, and bowel changes [8]. When radiation is localized to the head and neck region, patients may experience additional complications such as sore throat, xerostomia (dry mouth), dysphagia, and altered taste perception [9].

A particularly significant complication is oral mucositis, a painful inflammatory condition affecting the mucous membranes of the mouth [10]. It occurs in approximately 30-60% of head and neck cancer patients receiving radiation therapy [11]. This condition is especially prevalent due to the high turnover rate of the stratified squamous epithelium that lines the oral cavity, rendering these cells highly susceptible to radiation-induced injury. The World Health Organization (WHO) classifies oral mucositis into four distinct grades based on severity and clinical presentation. Grade 0 represents normal and healthy mucosa; Grade 1 identifies soreness/erythema localized to a mucosal spot; Grade 2 includes erythema/and ulcers, with the ability to eat solid foods; Grade 3 showcases ulcers and requires a liquid diet; and Grade 4 is categorized as where alimentation is not possible for the patient [10]. Although there is no definitive cure for oral mucositis, treatment focuses on managing symptoms and preventing secondary complications [12]. Strategies include the use of topical analgesics, meticulous oral hygiene, and nutritional support. Nevertheless, there is limited data on the natural resolution of mucositis and the return to healthy mucosal integrity [13].

This systematic review aims to evaluate current preventive measures for radiation-induced oral mucositis in patients with head and neck cancer. Specifically, this review will assess the effectiveness of various interventions by examining the incidence of oral mucositis, changes in WHO grading severity, and complications associated with each approach.

## Review

Methods 

**Figure 1:**
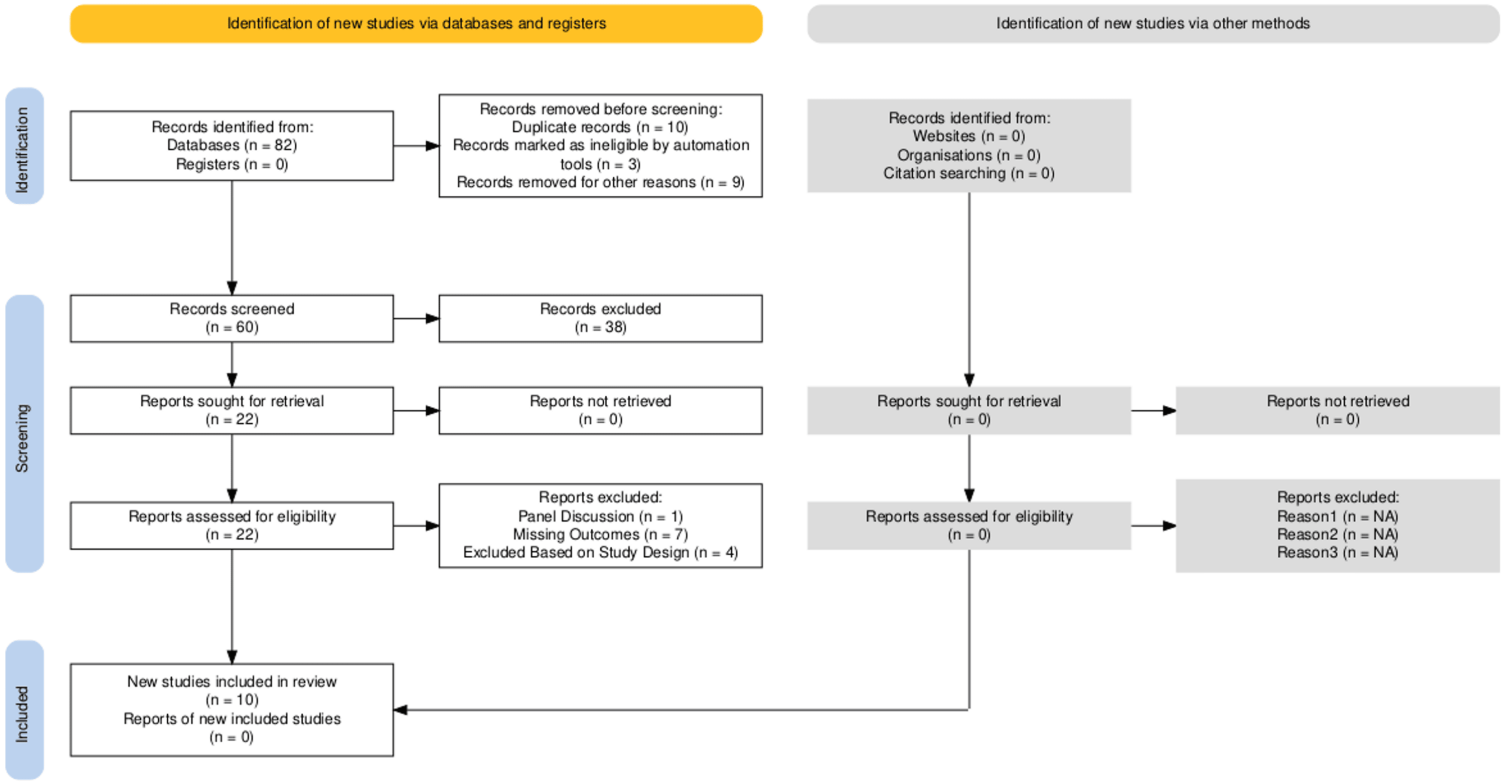
PRISMA Flowchart PRISMA: Preferred Reporting Items for Systematic Reviews and Meta-Analyses

**Figure 2:**
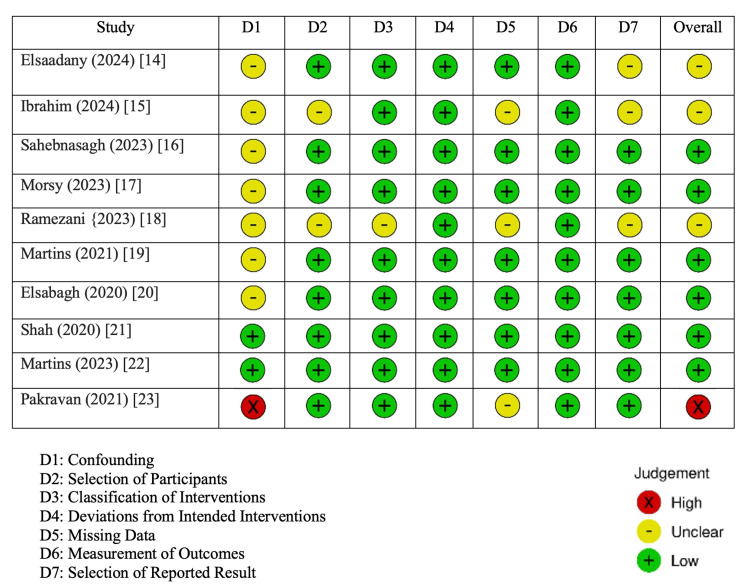
Cochrane Risk of Bias Assessment Image Credit: Raj Patel [14-23]

For the development of this systematic review, a literature search using Medical Literature Analysis and Retrieval System Online (MEDLINE) (PubMed) and Excerpta Medica Database (EMBASE) was performed to locate clinical literature on this topic from the index’s inception to October 2024 (Figure 1). Due to the abundant literature on oral mucositis (OM), the search strategies consisted of controlled vocabulary and keywords describing radiation-induced oral mucositis in specifically head and neck patients. The specific keywords that were utilized were: oral mucositis, radiation-induced oral mucositis, head and neck cancer radiation, and radiation therapy side effects. The inclusion criteria of this systematic review entailed only head and neck cancer patients who developed OM due to radiation therapy. Additionally, there was a requirement for specific outcome measurements that the study utilized to assess the progression of the OM symptoms. This entails structured pre- and post-operation measurements to allow for the proper analysis of each intervention. The team ensured that included articles included proper patient demographic data and reported complications and adverse effects. Studies were excluded if the cancer of interest in the paper was not of head and neck origin. Studies where OM was not induced by radiation therapy were also excluded. Additionally, if there were no/missing outcome measurements, the studies were not included due to a lack of analysis on their efficacy. Case reports, discussion panels, literature reviews, meta-analyses, and abstracts were excluded from this study. A risk of bias assessment was conducted to determine the validity of the extracted papers (Figure 2). There were 10 total papers extracted for this analysis (Table 1), and the mechanism of action of each intervention was identified (Table 2). 

**Table 1:** Final Included Studies

Study	Intervention Type	N
Elsaadany (2024) [14]	Rebamipide and Benzydamine	49 and 50
Ibrahim (2024) [15]	Glutamine	50
Sahebnasagh (2023) [16]	Zinc and Polyherbal Mouthwash	67
Morsy (2023) [17]	Omega-3 Nanoemulgel	34
Ramezani (2023) [18]	Oral and Topical Curcumin	37
Martins (2021) [19]	Photobiomodulation	48
Elsabagh (2020) [20]	Melatonin	40
Shah (2020) [21]	Curcumin Mouth Wash	8
Martins (2023) [22]	Mucoadhesive Phytomedicine	52
Pakravan (2021) [23]	Licorice Mucoadhesive Film	60

**Table 2:** Mechanisms of Action of Interventions Used in the Management of Oral Mucositis ATP: adenosine triphosphate; TNF-α: tumor necrosis factor-alpha

Study	Intervention Type	Mechanism of Action
Elsaadany (2024) [14]	Curcumin Mouth Wash	Curcumin mouthwash mitigates oral mucositis primarily by inhibiting the NF-κB signaling pathway, thereby suppressing transcription of pro-inflammatory cytokines and reducing mucosal inflammation.
Ibrahim (2024) [15]	Mucoadhesive Phytomedicine	Mucoadhesive phytomedicines alleviate oral mucositis primarily by forming a protective bioadhesive barrier on the mucosa while delivering plant-derived anti-inflammatory and antioxidant compounds that suppress NF-κB signaling and neutralize reactive oxygen species, promoting mucosal healing.
Sahebnasagh (2023) [16]	Glutamine	Glutamine alleviates oral mucositis primarily by serving as a fuel source for rapidly dividing mucosal epithelial cells, while also inhibiting NF-κB activation and reducing pro-inflammatory cytokines, thereby enhancing mucosal repair and reducing inflammation.
Morsy (2023) [17]	Rebamipide and Benzydamine	Rebamipide mitigates oral mucositis primarily by upregulating mucosal prostaglandin E2 synthesis and scavenging free radicals, which enhances mucosal defense, reduces inflammation, and promotes epithelial healing. Benzydamine acts by inhibiting pro-inflammatory cytokine production (e.g., TNF-α, IL-1β) and stabilizing cell membranes, providing local analgesic, anti-inflammatory, and antimicrobial effects to reduce mucosal pain and inflammation in oral mucositis.
Ramezani (2023) [18]	Zinc and Polyherbal Mouthwash	Zinc and polyherbal mouthwash alleviate oral mucositis by promoting mucosal epithelial cell regeneration and collagen synthesis, stabilizing cell membranes, inhibiting NF–κB–mediated inflammatory cytokine production, scavenging reactive oxygen species, and reducing microbial colonization to enhance healing and reduce inflammation.
Martins (2021) [19]	Omega-3 Nanoemulgel	Omega-3 nanoemulgel alleviates oral mucositis by enhancing localized delivery of eicosapentaenoic acid and docosahexaenoic acid, which inhibit COX–2–mediated prostaglandin E2 synthesis, reduce pro-inflammatory cytokines, and promote mucosal cell membrane repair and regeneration.
Elsabagh (2020) [20]	Oral and Topical Curcumin	Oral and topical curcumin alleviate oral mucositis by inhibiting the NF-κB signaling pathway, which reduces pro-inflammatory cytokine production, scavenges reactive oxygen species to decrease oxidative stress, and promotes mucosal epithelial cell proliferation and healing.
Shah (2020) [21]	Photobiomodulation	Photobiomodulation (PBM) alleviates oral mucositis primarily by stimulating mitochondrial cytochrome c oxidase, which increases ATP production, modulates reactive oxygen species, and activates transcription factors that reduce inflammation and promote tissue regeneration.
Martins (2023) [22]	Melatonin	Melatonin mitigates oral mucositis primarily by inhibiting NF-κB activation, thereby reducing pro-inflammatory cytokine expression (e.g., TNF-α, IL-1β) while also scavenging reactive oxygen species and enhancing antioxidant enzyme activity to protect and repair mucosal tissues.
Pakravan (2021) [23]	Licorice Mucoadhesive Film	Licorice mucoadhesive film mitigates oral mucositis primarily by delivering glycyrrhizin directly to ulcerated mucosa, where it inhibits NF-κB-mediated cytokine production and reduces COX-2 expression, while the mucoadhesive matrix ensures prolonged contact for sustained anti-inflammatory and epithelial-healing effects.

Results 

The analysis of OM development across various interventions reveals that the treatments assessed by Elsaadanya et al., specifically rebamipide and benzydamine, exhibited the highest efficacy (Table 3) [14]. These treatments resulted in significantly lower rates of OM, with only 40.5% and 59.5% of patients affected, respectively. In contrast, all patients in the studies by Ibrahim et al., Sahebnasagh et al., Morsy et al., Ramezani et al., Martine et al., and Elsabagh et al. developed OM, indicating a lack of efficacy for those treatments [15-20]. Partial efficacy was observed in the studies by Shah et al. and Martins et al., where 75% and 78.8% of patients, respectively, developed OM (Table 3) [21,22].

**Table 3:** Incidence of Radiation-Induced Oral Mucositis

Study	Intervention Type	Oral Mucositis Developed	
Elsaadany (2024) [14]	Rebamipide and Benzydamine	15/37 and 22/37	40.5% and 59.5%
Ibrahim (2024) [15]	Glutamine	50/50	100%
Sahebnasagh (2023) [16]	Zinc and Polyherbal Mouthwash	67/67	100%
Morsy (2023) [17]	Omega-3 Nanoemulgel	34/34	100%
Ramezani (2023) [18]	Oral and Topical Curcumin	48/48	100%
Martins (2021) [19]	Photobiomodulation	37/40	100%
Elsabagh (2020) [20]	Melatonin	60/60	100%
Shah (2020) [21]	Curcumin Mouth Wash	6/8	75%
Martins (2023) [22]	Mucoadhesive Phytomedicine	41/52	78.8%
Pakravan (2021) [23]	Licorice Mucoadhesive Film	---	---

The WHO scale refers to a system used to assess various health-related outcomes, including treatment effects in clinical settings. Specifically, the WHO Oral Toxicity Scale (also known as the WHO Oral Mucositis Scale) is often used to grade the severity of OM, a common side effect of cancer treatments such as radiation and chemotherapy. 

The WHO Oral Toxicity Scale measures the severity of OM on a scale from zero to four, where zero: no mucositis (normal oral health); one: soreness/erythema (mild symptoms); two: erythema, ulcers (but able to eat solid food); three: ulcers, requires a liquid diet (significant impairment); four: severe mucositis, requiring alimentation through intravenous feeding (life-threatening or disabling condition).

A comparison of the pre- and post-treatment WHO scale ratings for the severity of OM shows that Morsy et al. had the smallest difference, with a change of 0.76 (Table 4) [17]. Intermediate differences were observed in Shah et al. (+1.08), Elsaadanya et al. (rebamipide, +1.67), Martins et al. (+1.8), and Elsabagh et al. (melatonin, +1.5) (Table 4) [14,16,19,20,21]. The largest differences were reported by Elsaadany et al. (benzydamine, +2.18), Martins et al. (+2.22), and Sahebnasagh et al. (zinc and herbal, +2.71 and +2.58) (Table 4) [14,16,22]. Lastly, some studies showed a decrease in WHO scale ratings due to the presence of OM in the pretreatment phase. Ibrahim et al. reported a decrease of -1.5, Ramezani et al. showed decreases of -0.9 (wash) and -1.3 (oral), and Pakravani et al. recorded a decrease of -1.44 (Table 4) [15,18,23].

**Table 4:** WHO Scale (Pre- and Post-treatment) Δ: difference between post and pre-treatment numbers

Study	Intervention Type	N	Pre-treatment	Post-treatment	Δ
Elsaadany (2024) [14]	Rebamipide and Benzydamine	49 and 50	Rebamipide = 0, Benzydamine = 0	Rebamipide = 1.67, Benzydamine = 2.18	Rebamipide = +1.67, Benzydamine = +2.58
Ibrahim (2024) [15]	Glutamine	50	2.5	1	-1.5
Sahebnasagh (2023) [16]	Zinc and Polyherbal Mouthwash	67	Zinc = 0, Herbal = 0	Zinc = 0, Herbal = 2.8	Zinc = +2.71, Herbal = +2.58
Morsy (2023) [17]	Omega-3 Nanoemulgel	34	0	0.76	
Ramezani (2023) [18]	Oral and Topical Curcumin	37	Wash = 2, Oral = 3	Wash = 0.9, Oral = 1.3	Wash = -0.9, Oral = -1.3
Martins (2021) [19]	Photobiomodulation	48	0	1.8	+1.8
Elsabagh (2020) [20]	Melatonin	40	0	1.5	+1.5
Shah (2020) [21]	Curcumin Mouth Wash	8	0	+2.22	+1.08
Martins (2023) [22]	Mucoadhesive Phytomedicine	52	0	-1.5	+2.22
Pakravan (2021) [23]	Licorice Mucoadhesive Film	60	2.37	0.93	-1.44

Seven studies reported no complications associated with the interventions used: Ibrahim et al, Sahebnasagh et al., Ramezani et al., Elsabagh et al., Martins et al, Shah et al, Martins et al. (melatonin), and Pakravan et al. (Table 5). Martins et al. observed a complication rate of 2.9% (one out of 34 patients) due to a bitter taste from the omega-3 nanoemulgel intervention (Table 5). Elsaadany et al. reported a 12.5% (one out of eight patients) complication rate of burning sensation from the curcumin mouthwash (Table 5). In contrast, Morsy et al. documented the highest complication rates: 20% (10 out of 50 patients) for rebamipide and 28% (14 out of 50 patients) for benzydamine, with 10 patients experiencing nausea and 14 reporting burning sensations (Table 5). The dosage and intervention course is detailed in Table 6.

**Table 5:** Complications

Study	Intervention Type	Rate		Descriptions
Elsaadany (2024) [14]	Curcumin Mouth Wash	1/8	12.5%	(1) Burning Sensations
Ibrahim (2024) [15]	Mucoadhesive Phytomedicine	0/52	0%	
Sahebnasagh (2023) [16]	Glutamine	0/50	0%	
Morsy (2023) [17]	Rebamipide and Benzydamine	10/50 and 14/50	20% and 28%	(10) Nausea and (14) Burning Sensation
Ramezani (2023) [18]	Zinc and Polyherbal Mouthwash	0/67	0%	
Martins (2021) [19]	Omega-3 Nanoemulgel	1/34	2.9%	(1) Bitter Taste
Elsabagh (2020) [20]	Oral and Topical Curcumin	0/37	0%	
Shah (2020) [21]	Photobiomodulation	0/48	0%	
Martins (2023) [22]	Melatonin	0/40	0%	
Pakravan (2021) [23]	Licorice Mucoadhesive Film	0/60	0%	

**Table 6:** Dosage and Intervention Course

Study	Intervention Type	Dosage and Course
Elsaadany (2024) [14]	Rebamipide and Benzydamine	5 mL every 3 h (six times daily), and the gargle should generally be used undiluted, only swish for 30 s to one min, and spit.
Ibrahim (2024) [15]	Glutamine	5 g of glutamine combined with 5 g of maltodextrin, three times daily, 30 minutes before every meal, starting one day before radiation therapy.
Sahebnasagh (2023) [16]	Zinc and Polyherbal Mouthwash	5 mL of mouthwash, three times daily for 60 seconds, starting one day before radiation therapy.
Morsy (2023) [17]	Omega-3 Nanoemulgel	1 gram of gel self-applied by the patient, twice daily, every 12 hours, for six weeks, starting one day before radiation therapy.
Ramezani (2023) [18]	Oral and Topical Curcumin	10 ml of freshly prepared mouthwashes three times a day or one capsule of SinaCurcumin® (Exir Nano Sina Company, Tehran, Iran) 40 once a day for up to 21 days.
Martins (2021) [19]	Photobiomodulation	The laser was configured to a wavelength of 660 nm (red laser), power of 25 mW, in continuous mode. The density of energy was 6.2 J/cm 2 for 10 seconds; the area of the spot is 0.04 cm2, and thus, the energy of approximately 0.25 J was deposited at each point. The total energy per day was approximately 15.25 J.
Elsabagh (2020) [20]	Melatonin	10 mg, 2 tablets once daily for 6 weeks, 30 minutes before sleeping.
Shah (2020) [21]	Curcumin Mouth Wash	10 mL of mouthwash thrice daily for seven days without mixing with water; to avoid eating or drinking for 30 min after mouth rinse; to bring the bottle in the next visit (after seven days); not to skip the dosage; to avoid the use of any other oral gel or mouthwash.
Martins (2023) [22]	Mucoadhesive Phytomedicine	A red laser (660 nm), with a power of 25 mW and deposited energy of approximately 0.25 J per point, for 10 seconds, or 6.2 J/cm2, the daily energy applied was approximately 15.25 J. T.
Pakravan (2021) [23]	Licorice Mucoadhesive Film	Patients received a mucoadhesive film containing 30 mg of licorice extract, 0.1% triamcinolone acetonide, or placebo, applied to the upper lip mucosa four times daily (after each meal and at bedtime) for up to four weeks or until oral mucositis resolved.

Discussion 

Patients with head and neck cancer encounter a diverse therapeutic landscape, with radiation therapy being a common treatment modality. However, those undergoing radiation therapy must be informed of its associated side effects, one of the most prevalent being OM [1,2]. OM is an inflammatory condition affecting the mucous membranes of the mouth, often resulting in painful ulcers and sores [9]. Symptoms of OM include redness, swelling, and inflammation of the oral mucosa, leading to painful sores or ulcers. Other common symptoms are difficulty swallowing, dry mouth, sensitivity to spicy or acidic foods, and, in severe cases, bleeding or increased risk of infection [9]. Knowing the incidence of radiation-induced oral mucositis (RIOM), it is vital that there are treatments that patients can be given to reduce the incidence of OM. The purpose of this scoping review was to acknowledge proven treatments shown to reduce OM and compare their efficacies to one another to derive the most efficacious treatment.

To begin with, the incidence rates of OM were analyzed for each type of intervention. Notably, the study by Elsaadany et al. reported the lowest OM incidence, with 40.5% (15/37) of patients experiencing OM with oral rebamipide and 59.5% (22/37) with oral benzydamine (Table 3) [14]. In comparison, Shah et al.'s curcumin mouthwash and Martins et al.'s mucoadhesive phytomedicine demonstrated intermediate OM development, with incidence rates of 75% (6/8) and 78.8% (41/52), respectively (Table 3) [21,22]. On the other hand, the majority of the other interventions resulted in 100% OM development, as shown in Table 3. It is important to note that OM development is graded on a spectrum according to the WHO scale. While studies by Ibrahim et al., Sahebhasagh et al., Morsy et al., Ramezani et al., Martins et al., and Elsabagh et al. reported a 100% incidence of OM, the severity of the condition can vary and may correspond to a lower WHO scale grade [15-20]. In theory, though studies have all of their patients developing OM, the OM symptoms they are experiencing can be clinically minimal. 

Analyzing the percentage of patients who developed OM alone does not provide a comprehensive measure of the effectiveness of preventive treatments. To assess the true efficacy of each intervention, it is essential to consider outcome measures, which offer more concrete evidence of treatment results. The WHO scale, commonly used across the studies, was a primary outcome measure. This scale quantifies the progression of OM symptoms, making it a reliable tool for comparing results across different studies [11].

Most studies that employed the WHO scale used a pretreatment score of zero, establishing a baseline for the severity of OM symptoms before treatment. These studies included Shah et al., Martins et al. (mucoadhesive phytomedicine), Elsaadany et al., Sahebnasagh et al., Morsy et al., Martins et al. (photobiomodulation), and Elsabagh et al., all of which reported pretreatment WHO scores of zero [14,16,17,19-22]. This indicates that none of the patients had pre-existing symptoms of OM before treatment. The purpose of comparing the pre- and post-treatment WHO scores is to identify which interventions resulted in the smallest increase in symptom severity, thereby demonstrating their effectiveness in preventing or minimizing OM.

Among the interventions tested, Morsy et al.’s omega-3 nanoemulgel showed the smallest increase in the WHO score, with a difference of only +0.76 (Table 4) [17]. This suggests that patients treated with omega-3 nanoemulgel developed mild symptoms, such as soreness or erythema (redness), indicative of radiation-induced OM. The dosage and timing of administration were 1 gram of omega-3 nanoemulgel self-applied by the patient twice daily every 12 hours for six weeks, starting one day before radiation therapy (Table 6). Following this, Martins et al.’s photobiomodulation, Elsabagh et al.’s melatonin, Shah et al.’s curcumin mouthwash, and Elsaadany et al.’s rebamipide showed moderate increases in the WHO scale, with differences of +1.8, +1.5, +1.08, and +1.67, respectively (Table 4) [14,19-22]. These scores correspond to mild to moderate symptoms, ranging from soreness and erythema to the presence of ulcers. 

In contrast, studies that showed the largest increases in the WHO scale between pre- and post-treatment were Martins et al. (mucoadhesive phytomedicine), Elsaadany et al. (benzydamine), and Sahebnasagh et al. (zinc and polyherbal mouthwash), with scores of +2.22, +2.18, +2.17, and +2.58, respectively (Table 4) [14,16,19]. These larger differences indicate more severe OM symptoms, such as ulcers that significantly impair food intake and regular oral function. Considering these results, Morsy et al.’s omega-3 nanoemulgel stands out as the most effective treatment in preventing OM, as it resulted in clinically mild symptoms with minimal progression [17].

In contrast, the studies by Ibrahim et al., Ramezani et al., and Pakravan et al. involved patients who already had preexisting OM [15,18,23]. In these cases, it is crucial to evaluate whether the treatments reduced the severity of OM symptoms or, conversely, caused an exacerbation. For example, Ibrahim et al.’s glutamine oral pill led to a reduction of 1.5 points on the WHO scale, indicating that patients progressed from erythema and possible ulcers to less severe symptoms of soreness and erythema (Table 4) [15]. Similarly, Pakravan et al.’s licorice mucoadhesive film showed a WHO score decrease of -1.44, suggesting that patients with ulcers experienced a reduction in severity of soreness and erythema [23].

Finally, Ramezani et al.’s oral and topical curcumin mouthwashes resulted in decreases of 0.9 and 1.7 on the WHO scale, respectively (Table 4) [18]. The topical curcumin mouthwash had the greatest reduction of the three treatments, improving symptoms from ulcers that impaired oral health to mild soreness and erythema [20]. Though all patients in this study developed OM, the reduction in the WHO scale is worth noting. Though this study cannot be fully compared to other studies where there were no preexisting OM symptoms, these results suggest that curcumin may be a particularly effective treatment for patients with preexisting OM. In conclusion, topical curcumin appears to be a promising preventive and therapeutic treatment for OM, particularly in patients with preexisting symptoms. However, among the treatments studied, omega-3 nanoemulgel demonstrated the most consistent and effective results in preventing the development and progression of OM [19].

It is important to note that while Morsy et al. observed that all patients (34/34) developed OM, the severity of the condition was the lowest, with a WHO scale score increase of just +0.76 (Table 3) [17]. This indicates that, although every patient experienced some degree of erythema and soreness during radiation therapy, the symptoms were relatively mild. Given its ability to inhibit the progression of OM, this treatment should be considered by practicing dentists as a preventive option for patients undergoing radiation therapy. In contrast, Elsaadany et al.’s rebamipide demonstrated the lowest incidence of OM (40.5%), yet the difference in WHO scale scores between pre- and post-treatment was moderate, with a +1.67 increase (Table 3) [14]. This suggests that while 40.5% of patients experienced progression from mild soreness and erythema to more severe symptoms, such as ulcers, the impact on feeding and oral health remained minimal. Ultimately, it is the dentists’ responsibility to present these treatment options to patients, along with the respective incidence rates and severity of OM progression associated with each intervention. Providing this information will help patients make informed decisions when selecting a preventive treatment for OM as part of their head and neck cancer care plan.

After analyzing the outcome measures, the complications associated with each intervention were also considered. Morsy et al.’s omega-3 nanoemulgel exhibited a very low complication rate of 2.9%, with only one out of 34 patients reporting a bitter taste (Table 5) [17]. Given the positive outcomes discussed earlier, this low complication rate further supports the overall efficacy of this treatment. In contrast, Elsaadany et al. reported higher complication rates with rebamipide and benzydamine. 25% of patients (10/50) on rebamipide experienced nausea, and 28% (14/50) on benzydamine developed a burning sensation (Table 3) [14]. These relatively high complication rates should be carefully considered when recommending these treatments, as they may diminish their perceived efficacy and impact patient comfort. Finally, Shah et al.’s curcumin mouthwash had an intermediate complication rate of 12.5%, with one out of eight patients reporting a burning sensation (Table 5) [21]. However, due to the small sample size, this complication rate is not sufficient to draw firm conclusions about its overall efficacy or tolerability.

## Conclusions

Omega-3 nanoemulgel emerges as the most effective treatment for reducing OM in head and neck cancer patients undergoing radiation therapy. Its demonstrated ability to minimize symptom progression, with the smallest increase in WHO scale scores (+0.76), indicates its superior efficacy in preventing severe OM symptoms. Additionally, the treatment’s low complication rate of just 2.9%, with only a mild bitter taste reported by a single patient, highlights its excellent safety profile. In comparison, other treatments, such as rebamipide and curcumin mouthwash, showed higher complication rates and more moderate reductions in OM severity, making omega-3 nanoemulgel the preferred option. Given its proven effectiveness, minimal side effects on daily oral health, omega-3 nanoemulgel should be strongly considered as a first-line preventive treatment for OM in this patient population.
